# Dendritic cell-based vaccination in metastatic melanoma patients: Phase II clinical trial

**DOI:** 10.3892/or.2012.1956

**Published:** 2012-08-07

**Authors:** CHIE OSHITA, MASAKO TAKIKAWA, AKIKO KUME, HARUO MIYATA, TADASHI ASHIZAWA, AKIRA IIZUKA, YOSHIO KIYOHARA, SHUSUKE YOSHIKAWA, RYUJI TANOSAKI, NAOYA YAMAZAKI, AKIFUMI YAMAMOTO, KAZUTOH TAKESAKO, KEN YAMAGUCHI, YASUTO AKIYAMA

**Affiliations:** 1 Division of Immunotherapy, Shizuoka Cancer Center Research Institute, Shizuoka 411-8777; 2 Department of Dermatology, Shizuoka Cancer Center Hospital, Shizuoka 411-8777; 3 Division of Pathology and Clinical Laboratory of Medicine, National Cancer Center Hospital, Tokyo 104-0045; 4 Department of Dermatology, National Cancer Center Hospital, Tokyo 104-0045; 5 Department of Dermatology, Saitama Medical University, Saitama 350-0495; 6 Biotechnology Research Laboratories, Takara Bio Inc., Ltd., Shiga 520-2193, Japan

**Keywords:** dendritic cell, immunotherapy, metastatic melanoma, HLA-A24, overall survival

## Abstract

Metastatic and chemoresistant melanoma can be a good target of immunotherapy because it is an intractable cancer with a very poor prognosis. Previously, we tested a dendritic cell (DC)-based phase I vaccine, and confirmed that it was safe. In the present study, we performed a phase II trial of a DC vaccine for metastatic melanoma patients with mainly the HLA-A24 genotype, and investigated the efficacy of the vaccine. Twenty-four patients with metastatic melanoma were enrolled into a phase II study of DC-based immunotherapy. The group included 19 HLA-A24-positive (A^*^2402) patients and 3 HLA-A2-positive (A^*^0201) patients. The protocol for DC production was similar to that in the phase I trial. Briefly, a cocktail of 5 melanoma-associated synthetic peptides (gp100, tyrosinase, MAGE-A2, MAGE-A3 and MART-1 or MAGE-A1) restricted to HLA-A2 or A24 and KLH were used for DC pulsing. Finally, DCs were injected subcutaneously (s.c.) into the inguinal region in the dose range of 1–5×10^7^ per shot. The DC ratio (lin-HLA-DR^+^) of the vaccine was 38.1±13.3% and the frequency of CD83^+^ DCs was 25.7±20.8%. Other parameters regarding DC processing were not different from phase I. Immune response-related parameters including the ELISPOT assay, DTH reaction to peptide or KLH, DC injection numbers were shown to be related to a good prognosis. The ELISPOT reaction was positive in 75% of the patients vaccinated. The increase of anti-melanoma antigen antibody titer before vaccination was also shown to be a prognosis factor, but that post-vaccination was not. Based on immunohistochemical analysis, CD8 and IL-17 were not involved in the prognosis. Adverse effects of more than grade III were not seen. Overall survival analysis revealed a significant survival prolongation effect in DC-given melanoma patients. These results suggest that peptide cocktail-treated DC vaccines may be a safe and effective therapy against metastatic melanoma in terms of prolongation of overall survival time.

## Introduction

Metastatic and chemoresistant melanoma remains intractable and very difficult to treat. Based on the remarkable antitumor efficacy of dendritic cell (DC)-based vaccines in animal experiments (1,2), clinical trials of a DC-based immunotherapeutic approach have been conducted against mainly human leukocyte antigen (HLA)-A2^+^ advanced melanomas in Western counties (3–9). Since Nestle *et al*(3) first reported the efficacy of a DC vaccine against metastatic melanoma in a clinical trial, DC vaccines have become one of the main investigational therapeutic approaches against solid tumors. With regard to metastatic melanoma, it can be summarized that DC vaccine showed good safety and a low clinical response, but did not indicate a clear overall survival benefit (10–12). Moderate achievements were obtained in a phase I–II study, however unfortunately government-approved melanoma vaccines have yet to be developed because of the shortage of enrolled cases and a lack of double-blind, randomized (placebo-controlled) phase III trials.

Since sipuleucel-T (Provenge, Dendreon), an autologous cellular immunotherapy, was approved by the USA Food and Drug Administration (FDA), a significant benefit of DC-based vaccines on overall survival in metastatic castration-resistant prostate cancer patients had attracted much attention despite a low rate of clinical response (13–15). Like previous DC vaccine studies, in early phase trials, sipuleucel-T showed high safety, but a weak antitumor response which was not impressive compared with chemotherapeutic regimens. However, the last double-blind, placebo-controlled, multicenter phase III trial of the sipuleucel-T vaccine clearly demonstrated a significant survival benefit for metastatic prostate cancer.

Previously, we reported a phase I clinical trial of a DC-based vaccine against HLA-A24^+^ metastatic melanoma, and obtained several clinical responders (16). Based on these promising results, we have performed a phase II trial enrolling 24 metastatic melanoma patients. We summarize the results of a phase II non-randomized clinical trial and analyze various prognostic factors linked to an increase in overall survival.

## Materials and methods

### Patients and study design

Twenty-four patients with metastatic melanomas were enrolled in a phase II clinical trial of a peptide cocktail-pulsed DC-based vaccine from 2004 to 2010 approved by the Institutional Review Board (IRB) of Shizuoka Cancer Center, Japan. All patients gave written informed consent. Eligibility and exclusion criteria were similar to those for the previous phase I trial (16). The patients received the vaccine subcutaneously (s.c.) every week for 4 weeks, then once 2 weeks later and every month for 5 months. DCs were injected in the dose range of 1–5×10^7^/body/shot. Clinical response was rated as maximal through the DC vaccinations. The patients received up to 10 injections on the condition that one or more measurable lesion showed at lease a stable disease (SD) response and/or that an ELISPOT assay performed after 4 injections indicated a positive response against >1 melanoma-associated peptide. Adverse effects were evaluated according to the NCI Common toxicity criteria. Measurable lesions and clinical responses were evaluated by RECIST (17).

With regard to overall survival, as a retrospective control, survival data from 37 patients with metastatic melanoma given best supportive care without a DC vaccine from 2004 to 2008 were utilized with approval by the IRB of Shizuoka Cancer Center.

### Preparation of the DC vaccine

The methods used to produce the DC vaccine were described previously (16). Briefly, monocyte-enriched fractions were separated from leukapheresis products using OptiPrep™, and cultured in the presence of granulocyte macrophage-colony-stimulating factor (GM-CSF) and interleukin (IL)-4 in X-VIVO15 serum-free medium. After 7 days, harvested cells were pulsed with a cocktail of 5 melanoma-specific synthetic peptides restricted to HLA-A2 or A24 and keyhole limpet hemocyanin (KLH). DC-enriched cells were washed and cryopreserved in a Cryocyte bag until used. The following peptides restricted to HLA-A2 or A24 were synthesized according to good manufacturing practice (GMP) standards by Multiple Peptide Systems, CA: HLA-A2: MART-1, gp100, tyrosinase, MAGE-A2, and MAGE-A3 and HLA-A24: gp100, tyrosinase, MAGE-A1, MAGE-A2 and MAGE-A3.

### Characterization of tumor specimens using RT-PCR and immunohistochemistry

Specimens of primary tumors or skin metastatic lesions were obtained from 12 patients in a phase II study. The expression of melanoma tumor antigens was investigated using RT-PCR. HLA-class I protein expression and the phenotypes of lymphocytes infiltrating the tumor site were examined using immunohistochemistry (IHC). The monoclonal antibodies (mAbs) against human HLA-class I (Hokudo Co., Ltd., Sapporo, Japan), CD8 (Thermo Scientific, Flemont, CA), Foxp3 (Abcam, Cambridge, MA) and IL-17 (Abcam) were all purchased commercially.

### Immunological monitoring

The ELISPOT assay, intracellular cytokine staining using anti-human interferon (IFN)-λ and anti-IL-4 antibodies and delayed-type hypersensitivity (DTH) skin tests were described previously (16). Briefly, the HLA-A2 or A24 peptide cocktail solution diluted to a dose of 5 μg/ml and KLH were injected intradermally into the forearm for DTH measurements after 48 h.

### Serum autoantibody against melanoma antigens

The ELISA for detecting human antibodies and the control reaction system were described previously (18). MAGE antigen proteins with a glutathione S-transferase (GST) tag were all purchased from Abnova Corp., Taipei, Taiwan. Recombinant MAGE-A1 and -A2 were full-length constructs and MAGE-A3 was a fragment, comprising amino acids 1–135. Briefly, recombinant human MAGE antigens were added to a 96-well microplate, and sequentially diluted rabbit anti-human IgG antibody was added. After blocking with 3% bovine serum albumin (BSA), diluted patient serum (before or after vaccine) or human IgG at 100 μg/ml was added to antigen-coated wells or anti-human IgG-coated wells, respectively. After incubation, sheep horse-HRP-conjugated anti-human IgG antibody, then substrate solution was added and the absorbance at 450 nm was read. A serum antibody index was calculated using the control calibration curve as follows; index = [capture antibody dose (μg/ml)/control OD × sample OD/dilution ratio of sera].

### Statistical analysis

The overall survival of metastatic melanoma patients was examined by comparing differences in mean survival time (MST) via the Kaplan-Meier method. A comparative analysis of survival times between groups was then performed using the log-rank test. Values of P<0.05 were considered statistically significant.

## Results

### Patient characteristics

Patient details are summarized in Table I. Most of the patients were in good performance status (PS), were HLA-A24^+^ and had received prior chemotherapy. As to the number of target lesions, half of the patients had >2 metastatic lesions, the average being 1.7±0.7 (Table II).

### DC processing and characterization

The mean number of peripheral blood mononuclear cells (PBMCs) collected by apheresis in phase II patients was 8.5±2.5×10^9^. The CD14 frequency increased from 19.5±10.6 to 44.7±15.3% after OptiPrep density-gradient centrifugation. The mean percentage of DCs rated as lin^−^CD11c^+^HLA-DR^+^ was 38.1±13.3%, not different from that in the phase I trial (data not shown). The frequencies of the DC marker including CD83, CD80, CD86, DC sign, DEC205 and CMRF56, and DC1/DC2 ratio did not differ from the previous report either (data not shown).

### Clinical responses and adverse effects

Among 24 cases, 1 case of partial remission (PR), 7 cases of SD and 16 cases of progressive disease (PD) were verified (Table II). The injected mean DC number was up to 5.0×10^7^/body at a maximum, averaging 2.4×10^7^/body/shot. DC injection times were 10.5 on average, and 15 cases were completed with 10 injections. No significant side effects of more than grade III were seen.

### Immunological monitoring

Eighteen of 24 cases (75%) showed positive ELISPOT reactions against all melanoma antigen-related peptides (Table III). Six exhibited reaction against >3 peptides. As to the Th1/Th2 balance after vaccines, 12 of 19 evaluable cases had a ratio of >1, which indicated a shift to Th1 development. With regard to skin tests, DTH reactions against peptide-pulsed DC and KLH were detected in 41 and 64%, respectively, of vaccinated patients (Table II).

### Serum antibodies against melanoma antigens

**Figure 1:**
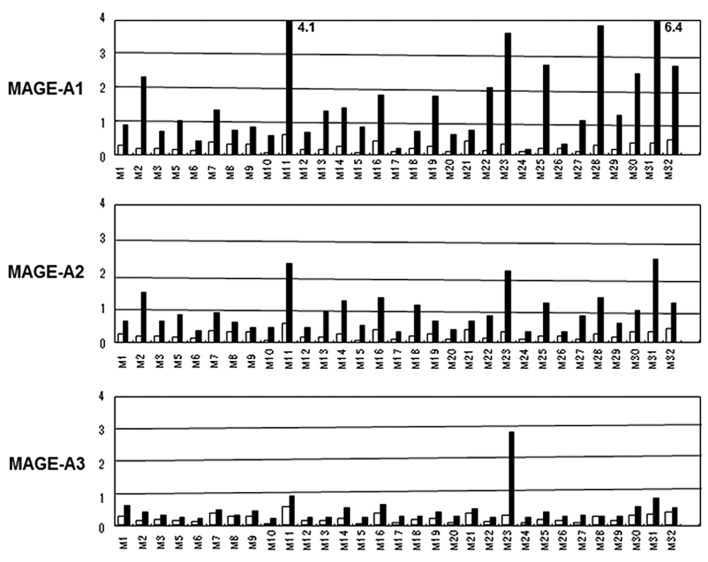
Serum autoantibody against MAGE antigens in melanoma patients before the vaccination. Sera derived from 31 evaluable cases in the phase I and II trials were analyzed. The ELISA for human antibody detection and the control reaction system were described previously. Recombinant GST-tagged MAGE proteins were used as antigens. Open column, GST alone; closed column, GST-tagged MAGE proteins for antigen.

Prior to DC vaccines, positive cases of anti-MAGE-A1, -A2, -A3 and tyrosinase antibodies numbered 15, 10, 1 and 9, respectively among 31 evaluable cases from the phase I and II trials. The positive rate of anti-melanoma antigens antibody was 54.8% (Fig. 1, Table IV). On the other hand, the positive rate of any antibody after vaccination was not high (40.7%) compared with before the vaccine. The index ratio means the Ab index after vaccine/before vaccine.

### Characterization of tumor tissues

**Figure 2:**
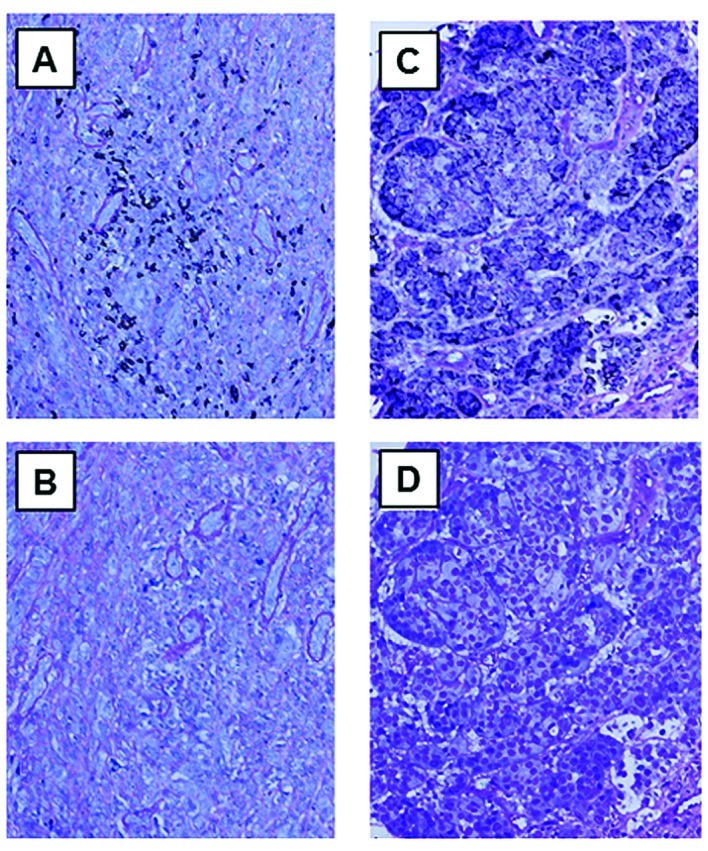
Immunohistochemical analysis of CD8 and IL-17 expression in melanoma tumors. (A) and (B) tumor tissue from MEL-2; (C) and (D) tumor tissue from MEL-11. (A) CD8 staining, (C) IL-17 staining, (B) and (D) isotype control antibody staining. The counter-staining was performed with the Giemsa stain. Magnification, ×100.

IHC was performed using tumor tissue sections derived from 22 melanoma patients of phase I and II trials. Fifteen tumor tissues in which melanoma antigen expression was analyzed using RT-PCR, showed >2 antigens (Table III, phase I data not shown). Meanwhile, IHC analysis demonstrated that most (82%) of the 22 evaluable tumor tissue specimens showed HLA-class I expression, and that CD8 and IL-17 positive staining was seen in 60% and 53%, respectively (Table V, Fig. 2).

### Overall survival analysis

Based on survival data from all metastatic melanoma patients including these of the phase I and II study, various clinical, immunological and DC-processing-related parameters were analyzed in terms of the relationship to the prognosis of melanoma patients.

**Figure 3:**
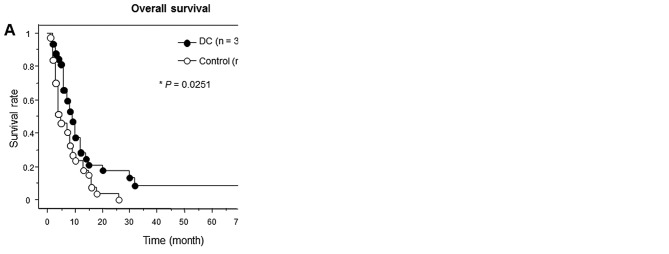
Survival time in melanoma patients given the DC vaccine Thirty-three metastatic melanoma patients enrolled in phase I–II trials were analyzed. Survival data derived from 37 cases of melanoma given best supportive care without DC vaccine were utilized as a control. (A) Survival analysis of melanoma patients with and without the DC vaccine. ○, without DC (n=37); ●, with DC (n=33). (B) Survival analysis for high (≥2) and low (<2) ELISPOT scores. ○, score <2 (n=15); ●, score ≥2 (n=18). The difference was analyzed using the log-rank test. Values of P<0.05 were considered statistically significant.

First of all, overall survival analyses were performed between the vaccinated and non-vaccinated patients, and between high (≥2) and low (<2) ELISPOT score groups (Fig. 3). The ELISPOT score indicates the number of peptides with a positive cytotoxic T-cell (CTL) response. The vaccinated group and ELISPOT high score group demonstrated significantly longer mean survival times (13.6 M in vaccinate vs. 7.3 M in non-vaccinated; 21.9 M in high ELISPOT score vs. 8.1 M in low).

Second, DC processing-related parameters including injected DC numbers, DC ratio, and surface markers such as CD83 and CCR7 did not demonstrate any relationship to overall survival (Table VI). Third, various immunological parameters were analyzed in terms of survival prolongating effects (Table VII). The number of target lesions and the number of DC injections showed a significant correlation with survival time. Interestingly, the anti-MAGE-A1 antibody titer before the vaccination was shown to be a good prognostic factor, but anti-melanoma antigen antibody induction after vaccination was not. Immune response parameters such as ELISPOT, DTH reaction against peptide and KLH also showed a significant survival benefit. However, IHC parameters such as CD8 and IL-17 stain levels were not relevant to survival possibly due to the shortage of case numbers where a tumor specimen was obtained.

## Discussion

Many clinical trials of immunotherapy using DC-based vaccines against metastatic melanoma have been performed, since Nestle *et al*(3) reported the efficacy of a melanoma lysate or peptide-treated DC vaccine in 1998. In most trials, the number of registered cases was around 20 with mainly the HLA-A2 genotype, and only a few cases of clinical response [partial remission (PR) and complete remission (CR)] were reported. The difference of antigen source like synthetic peptide or tumor lysate and maturation status did not seem to be closely related to the clinical response (19). Additionally, a randomized phase III study of melanoma DC vaccines has not yet been performed based on early phase I, II studies. We have been testing a peptide-based DC vaccine against HLA-A24^+^ metastatic melanoma in phase I and II clinical trials. The ethnicity of HLA genotyping revealed a significant difference of CTL epitope sequence and immunological responses, which suggested that HLA-A24^+^-based immune response is unique and should be investigated more intensively.

In the present study, based on survival data from all metastatic melanoma patients from phase I and II trials, potential prognostic factors were investigated among various clinical, immunological and DC-processing-related parameters in terms of the prolongating effect on overall survival. Unfortunately, DC processing-related parameters did not show any effect on overall survival. Interestingly, the anti-MAGE-A1 antibody titer before the vaccination was shown to be a possible prognostic factor, but anti-melanoma antigen antibody induction after the vaccination was not. Recently, an autoantibody signature-based approach has been used to discover novel tumor antigens (20–22). Especially, in melanoma patient-derived serum, novel biomarkers involved in lymph node metastasis prediction were identified (23). As to MAGE antigens, Stockert *et al* reported that an autoantibody against MAGE-A1 was detected in only 3 of 234 cancer patients (24), which was a very low frequency compared with ours (48.4% in metastatic melanoma patients). Impressively, our study demonstrated that the anti-MAGE-A1 autoantibody was positively correlated with overall survival, which seems to be a novel observation. Meanwhile, the number of target lesions and immune response parameters such as ELISPOT, DTH reaction against peptide and KLH showed a prolongation effect on overall survival, which was reasonable because tumor load and immunological responses are known to be closely linked to prognosis in melanoma patients (3,8).

The infiltration of CD8^+^ and TH17 cells at the tumor site is reported to be closely involved in the prognosis of solid cancer patients (25–27). In our study, the positive rate of CD8 and IL-17 was 60 and 53%, respectively, in 15 resected tumors. However, a significant correlation to prognosis was not seen because of the small number of cases.

Since sipuleucel-T (Provenge) immunotherapy was approved by the FDA, DC-based cancer vaccine studies have been encouraged and enhanced to develop the advanced stage of clinical trials (28,29). As is the case with sipuleucel-T, there will be some problems with DC-based cancer vaccines. One is that the time for clinical evaluation might be too short to expect prolongation of survival time because the optimal immune response would have several weeks to operate and pass the cancer progression. A conventional clinical evaluation based on RECIST criteria is incompatible with overall survival benefit obtained only by the continual administration of vaccine despite clinical progression. To define the progression precisely in prostate cancer, the Prostate Cancer Working Group recently devised progression guidelines (30).

Very recently, studies of novel cancer vaccines like sipuleucel-T and MAGE-A3 and other long peptides with conjugation were activated at subclinical levels, which demonstrates the coming of a new era for cancer vaccines (31–33). The bottom line is that sequentially to the success of sipuleucel-T trials, more phase III randomized studies of specific peptide-pulsed DC vaccines should be performed. Additionally, a world-wide network of translational research facilities which can perform high-grade clinical immunotherapeutic research has to be constructed. These efforts could lead to more efficient cancer vaccines in the near future.

## Figures and Tables

**Table I:** Characteristics of metastatic melanoma patients in the phase II study.

Total no. enrolled	24
Age	58.2±12.2
Gender	
M	12
F	12
Performance status	
PS0	22
PS1	2
HLA-typing	
A2	3
A24	21
Previous therapy	
ST	1
CT	3
ST+CT	19
ST+CT+RT	1

ST, surgical therapy; CT, chemotherapy; RT, radiation therapy.

**Table II:** Phase II study of DC-based therapy against melanoma.

						DTH		
							
Case	Age	Gender	Measurable lesions	DC no. (times)	Side effects	DC	KLH	Response (ST)^a^
MEL-1	53	F	Liver	4.2×10^7^(10)	Fever (II)	+	+	SD (7.5)
MEL-2	50	F	Lung	5.2×10^7^(10)	ND	+	+	PD (7)
MEL-3	46	F	Lung	0.5×10^7^(11)	ND	+	+	SD (8.5)
MEL-4	62	M	Lung, liver, LN	7.3×10^7^(8)	Hepatic (I)	−	+	PD (6)
MEL-5	63	M	Lung, liver	3.1×10^7^(8)	Hepatic (II)	−	−	PD (6)
MEL-6	45	F	Lung	0.7×10^7^(10)	Leucopenia (II)	+	+	SD (30)
MEL-7	57	F	Lung	0.3×10^7^(10)	ND	−	+	PD (12)
MEL-8	68	M	Skin	0.6×10^7^(14)	ND	+	+	PR (12)
MEL-9	56	M	Lung, LN	NE (15)	Hepatic (I)	+	+	SD (32)
MEL-10	73	F	Lung, liver	NE (5)	ND	−	−	PD (3.5)
MEL-11	68	F	Liver, LN	NE (6)	ND	−	−	PD (5.5)
MEL-12	53	M	Skin, LN	NE (6)	Hepatic (II)	−	+	PD (9)
MEL-13	63	F	Liver, LN	0.6×10^7^(10)	ND	−	−	PD (12)
MEL-14	73	F	Lung	1.5×10^7^(3)	ND	ND	ND	PD (1.5)
MEL-15	32	M	Lung, LN	1.0×10^7^(10)	ND	−	+	PD (7)
MEL-16	62	F	Lung	1.1×10^7^(8)	ND	−	−	PD (6)
MEL-17	60	M	Lung, LN	1.2×10^7^(12)	Fever (I)	−	+	PD (10)
MEL-18	82	F	Nasal cavity	0.5×10^7^(10)	ND	−	−	PD (9.5)
MEL-19	63	M	Lung	0.9×10^7^(25)	Hepatic (I)	+	+	SD (60)
MEL-20	40	M	Lung, bone	1.7×10^7^(4)	ND	ND	ND	PD (3)
MEL-21	36	M	Lung	1.2×10^7^(20)	ND	+	+	PD (46)
MEL-22	63	F	LN	3.9×10^7^(6)	ND	+	+	PD (7.5)
MEL-23	70	M	Gingiva, lung	1.9×10^7^(20)	ND	−	−	SD (18.5)
MEL-24	59	M	Lung, skin	1.5×10^7^(13)	ND	−	−	PD (12.5)

a ST, overall survival time; NE, not evaluated; ND, not detected.

**Table III:** Immunological monitoring in melanoma patients (phase II).

Case	HLA typing	Tumor antigen expression^a^	HLA-class I expression	DC1/DC2 ratio	ELISPOT	Th1/Th2 balance^b^
MEL-1	A^*^2402	ND	ND	192	1 (MAGE1)	1.24
MEL-2	A^*^2404	5/5	+ to ++	7.6	0	NE
MEL-3	A^*^2402	ND	ND	149	3 (MAGE1-3)	1.3
MEL-4	A^*^2402	2/5	+ to ++	45.4	1 (Tyr)	0.83
MEL-5	A^*^2420	ND	ND	13.8	0	0.91
MEL-6	A^*^2402	2/5	++	104	3 (MAGE1-3)	1.12
MEL-7	A^*^2402	4/5	++	125	3 (MAGE1-3)	1
MEL-8	A^*^2402	4/5	++ to +++	435	2 (MAGE1,2)	NE
MEL-9	A^*^0201	ND	ND	18.1	0	1.36
MEL-10	A^*^2402	3/5	+	103	2 (MAGE1,2)	1.25
MEL-11	A^*^2402	3/5	+	24.1	2 (MAGE1,2)	0.79
MEL-12	A^*^2402	ND	++ to +++	35.0	1 (MAGE3)	1.17
MEL-13	A^*^2402	ND	ND	78.0	0	0.67
MEL-14	A^*^2402	5/5	+	8.8	0	ND
MEL-15	A^*^2402	3/5	++	7.2	4 (MAGE1-3,Tyr)	0.46
MEL-16	A^*^0201	ND	ND	10.4	1 (MAGE2)	1.25
MEL-17	A^*^0201	5/5	+	34.0	2 (gp100,MAGE2)	1.01
MEL-18	A^*^2402	ND	ND	28.6	3 (MAGE1-3)	1.45
MEL-19	A^*^2402	ND	ND	12.6	3 (MAGE1-3)	1.69
MEL-20	A^*^2402	ND	ND	73.8	ND	ND
MEL-21	A^*^2402	ND	ND	127	2 (MAGE1,2)	0.36
MEL-22	A^*^2402	4/5	+	16.3	1 (MAGE3)	ND
MEL-23	A^*^2402	ND	ND	2.2	2 (Tyr,MAGE3)	0.81
MEL-24	A^*^2402	2/5	+	6.3	1 (MAGE3)	1.81

a No. of positive antigens of 5 melanoma antigens (tyrosinase, gp100, MAGE1, 2, 3).

b Th1/Th2 balance shows the ratio of post-/pre-vaccine-Th1/Th2.

NE, not evaluated; ND, not done.

**Table IV:** Positive rate of serum autoantibody against melanoma antigens.

Antigens	MAGE-A1	MAGE-A2	MAGE-A3	Tyrosinase	Any antigen
Pre (Index >1)	15/31	10/31	1/31	9/27	17/31
Post (Index ratio^a^ >2)	4/19	4/27	5/25	7/27	11/27

a Index ratio means the antibody index after/before vaccine for melanoma antigens.

**Table V:** Immunohistochemical features of melanomas: phase I, II study.

Antigens	HLA-class I	CD8	Foxp3	IL-17
Melanoma tumors (positive %)	18/22 (82)	9/15 (60)	4/15 (27)	8/15 (53)

**Table VI:** Prognostic factors for melanoma DC vaccines-1.

Factors	Cases	Mean ± SD	Groups (MST)	Statistical analysis
DC nos. (×10^7^)	29	2.4±1.8	<2 (16.4) vs. ≥2 (15.5)	NS
DC ratio (%)	33	38.1±13.3	<40 (15.9) vs. ≥40 (15.3)	NS
DC1/DC2 ratio	33	107±129	<100 (12.6) vs. ≥100 (20.3)	NS
CD40 (%)	33	66.8±18.9	<70 (15.3) vs. ≥70 (15.9)	NS
CD83 (%)	33	25.7±20.8	<25 (14.5) vs. ≥25 (16.9)	NS
CD83^+^ DC no. (×10^6^)	33	5.5±6.4	<5 (15.4) vs. ≥5 (16.8)	NS
CCR7 (%)	33	29.1±21.8	<25 (14.3) vs. ≥25 (17.0)	NS

NS, statistically not significant.

**Table VII:** Prognostic factors for melanoma DC vaccines-2.

Factors	Cases	Mean ± SD	Groups (MST)	Statistical analysis
No. of target lesions	33	1.7±0.7	<2 (24.1) vs. ≥2 (8.6)	0.029^g^
No. of DC injections	33	9.8±5.5	<10 (6.2) vs. ≥10 (24.5)	<0.0001^h^
Anti-melanoma antigen Ab^a^ (Pre-DC)	31	1.6±1.4^b^	<1 (3.8) vs. ≥1 (22.9)	0.002^h^
Anti-melanoma antigen Ab (Post-DC)	31	11/27^c^ (35%)	neg. (16.9) vs. pos. (18.5)	NS
ELISPOT assay	33	1.5±1.1^d^	<2 (8.1) vs. ≥2 (21.9)	0.0125^g^
DTH (peptide or DC)	31	12/31^e^ (39%)	neg. (8.9) vs. pos. (28.3)	0.0105^g^
DTH (KLH)	31	18/31^f^ (58%)	neg. (8.2) vs pos. (22.4)	0.0244^g^
Th1/Th2 balance	29	1.2±0.6	≥1 (12.2) vs. >1 (24.1)	NS
CD8^+^ T cell in tumors	16	9/16 (56%)	neg. (6.5) vs. pos. (10.9)	NS
IL17 stain in tumors	16	8/16 (50%)	neg. (10.5) vs. pos. (7.5)	NS

a Anti-MAGE-A1 Ab

b antibody index

c ratio of Ab index (post-DC/pre-DC) >2

d no. of peptide with positive response

e positive DTH for peptide or DC and

f positive DTH for KLH.

g P<0.05,

h P<0.01.

NS, not significant.

## Abbreviations

DC: dendritic cell
HLA: human leukocyte antigen
FDA: food and drug administration
IRB: institutional review board
GM-CSF: granulocyte macrophage-colony-stimulating factor
IL: interleukin
KLH: Keyhole limpet hemocyanin
IHC: immunohistochemistry
CTL: cytotoxic T cell
DTH: delayed-type hypersensitivity
CR: complete remission
PR: partial remission
SD: stable disease
PD: progressive disease
ELISA: enzyme-linked immunosorbent assay
IFN: interferon
HRP: horseradish peroxydase
PS: performance status
PBMC: peripheral blood mononuclear cell
